# Discrimination Method of the Volatiles from Fresh Mushrooms by an Electronic Nose Using a Trapping System and Statistical Standardization to Reduce Sensor Value Variation

**DOI:** 10.3390/s131115532

**Published:** 2013-11-13

**Authors:** Kouki Fujioka, Nobuo Shimizu, Yoshinobu Manome, Keiichi Ikeda, Kenji Yamamoto, Yasuko Tomizawa

**Affiliations:** 1 Department of Molecular Cell Biology, Institute of DNA Medicine, the Jikei University School of Medicine, Tokyo 105-8461, Japan; E-Mails: kfujioka@jikei.ac.jp (K.F.); ikedak@jikei.ac.jp (K.I.); 2 Metric Science Group, Department of Data Science, the Institute of Statistical Mathematics, Tokyo 190-8562, Japan; E-Mail: nobuo@ism.ac.jp; 3 Core Research Facilities, the Jikei University School of Medicine, Tokyo 105-8461, Japan; E-Mail: manome@jikei.ac.jp; 4 National Center for Global Health and Medicine, Tokyo 162-8655, Japan; E-Mail: ykenji@hosp.ncgm.go.jp; 5 Department of Cardiovascular Surgery, Tokyo Women's Medical University, Tokyo 162-8666, Japan

**Keywords:** electronic nose, smell, mushrooms, humidity, alcohol, variation, z-score, standardization, flavor, Le Nez du Vin

## Abstract

Electronic noses have the benefit of obtaining smell information in a simple and objective manner, therefore, many applications have been developed for broad analysis areas such as food, drinks, cosmetics, medicine, and agriculture. However, measurement values from electronic noses have a tendency to vary under humidity or alcohol exposure conditions, since several types of sensors in the devices are affected by such variables. Consequently, we show three techniques for reducing the variation of sensor values: (1) using a trapping system to reduce the infering components; (2) performing statistical standardization (calculation of z-score); and (3) selecting suitable sensors. With these techniques, we discriminated the volatiles of four types of fresh mushrooms: golden needle (*Flammulina velutipes*), white mushroom (*Agaricus bisporus*), shiitake (*Lentinus edodes*), and eryngii (*Pleurotus eryngii*) among six fresh mushrooms (hen of the woods (*Grifola frondosa*), shimeji (*Hypsizygus marmoreus*) plus the above mushrooms). Additionally, we succeeded in discrimination of white mushroom, only comparing with artificial mushroom flavors, such as champignon flavor and truffle flavor. In conclusion, our techniques will expand the options to reduce variations in sensor values.

## 1. Introduction

Electronic noses have the benefit of obtaining smell information in a simple and objective manner, therefore, many applications have been developed for electronic noses, including quality control, the determination of freshness, classification, and the detection of microorganisms in fields such as foods and beverages, cosmetics, medicine and agriculture [1–7]. In particular, advances have been reported in the food area, such as the detection of differences in the quality of wheat products [8], differences in coffee aromas [9], changes in temperature and their corresponding changes in smell for frozen foods [10], and differences in the aroma of dried mushrooms [11].

As is the case for coffee and wine, mushroom aroma is utilized as a means of discerning the food value of mushrooms, so it has been analyzed using gas chromatography/mass spectrometry (GC/MS) systems (Table 1) [12–16]. Furthermore, characteristic aroma has also been used as a marker of mushroom discrimination [17,18]. The main components of mushroom aromas include a variety of alcohols, and in particular 1-octen-3-ol (mushroom alcohol or matsutake alcohol) is commonly detected in many varieties of mushrooms. However, the components of fresh mushrooms vary per their place of origin and can change depending on the period of time after collection and storage temperature. In a study about matsutake mushroom, the amount of the total aroma differed with the production area [19]. Additionally, the aroma was reduced by one half when stored at 27 °C for 66 h. Another study has reported that truffles, when stored for seven days, showed less change in aroma at +8 °C, comparing with +4 °C [20].

Previous studies on mushroom discrimination using several sensors have reported the classification of mushrooms in the same genus [11,21,22]. Miyashita *et al.* used organic semiconductor thin film sensors to discern the productive area by the volatiles of Japanese and Chinese fresh shiitake mushrooms (four Japanese varieties and three Chinese varieties), but they could not discern the area due to the high variations in the data [21]. Aside from fresh mushrooms, Pinalli *et al.* used three varieties of dried *Boletus* mushroom from three different production areas (Italy, Northern China, and Southern China), and conducted differential function analysis (DFA). They reported that compared to the Italian variety, they could distinguish the Northern Chinese variety and Southern Chinese variety with accuracies of 86.5% and 94.5%, respectively [11]. Also, Keshri *et al.* have reported that using principal component analysis (PCA) of volatile components from cultured mycelia, it was possible to discriminate between five of seven *Agaricus* species, but that some overlap was observed among samples [22].

These previous studies suggest that the following two problems should be solved in mushroom discrimination using sensors: (1) there are many variations in the measurement values due to unstable component conditions; (2) even among mushrooms of the same variety, sensor values are affected by the differences of components due to production area and species.

Assuming that these problems are solved—making it possible to accurately discriminating fresh mushrooms using sensors—these three merits may be provided. First, the development of a technology for discriminating mushrooms using aroma may result in the ability to discern toxic mushrooms. Although food poisoning from wild mushrooms has decreased, it is still reported today in many countries all over the world (Japan [23], Europe [24], the United States [25], *etc.*). If a simple discerning system is developed, it may lead to a reduction in these cases of food poisoning. Second, the discrimination of mushrooms using an electronic nose may be useful as information for consumer preferences or when a buyer is judging quality. Mushrooms have commercial value associated with their aromas. When investigating or presenting mushrooms through media where it is difficult to convey aroma-related information (TV, internet, magazines, *etc.*), the objective aroma information will be helpful. Third, discriminating mushrooms with sensors may lead to the development of detection devices. Currently, mushroom bed cultivation for truffles or matsutake is still difficult. The development of a sensor-based method that accurately detects these mushrooms may make it easier to discover them in field.

Therefore, in this study, we focused on the control of the variations of sensor values. To improve discrimination accuracy, we introduce three methods: (1) using a carbon graphite trap tube suitable for measuring non-polar components in order to reduce the influence of alcohol and water content; (2) statistical standardization (calculation of z-score) in order to reduce the impact of the total aroma amount (smell strength) so as to enable discrimination according to aroma quality; and (3) sensor selection for discrimination. With these techniques, we have attempted to discriminate six mushroom varieties: golden needle (*Flammulina velutipes*), eryngii (*Pleurotus eryngii*), hen of the woods (*Grifola frondosa*), white mushrooms (*Agaricus bisporus*), shiitake (*Lentinus edodes*), and shimeji (*Hypsizygus marmoreus*). Although excluding shimeji, the volatile components for the other five mushroom varieties have been revealed with GC/MS (Table 1), no study has reported sensor-based discrimination of these six fresh mushroom types. Additionally, we have investigated the possibility of discriminating mushrooms using correlation coefficients (*r*) with the artificial mushroom flavors champignon and truffle flavors.

## 2. Experimental Section

### 2.1. Fresh Mushroom Samples and Flavor Samples

**Figure 1.**
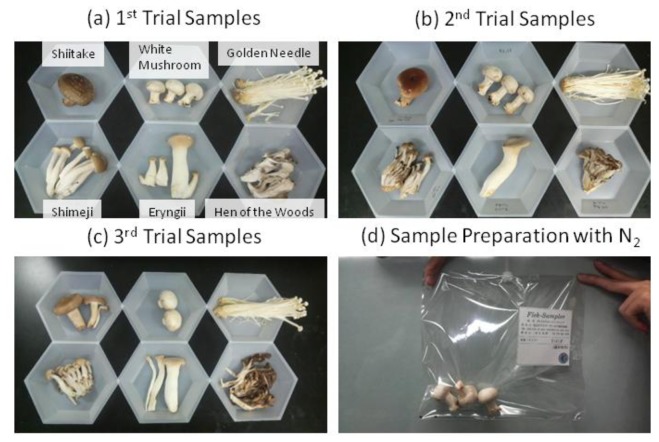
Approximately 30 g of mushrooms used for measurements (**a)**–(**c**) and the sample bag that was filled with dry nitrogen after placing the mushrooms inside (**d**).

The six fresh mushroom samples (each approximately 30 g) of shiitake, white mushrooms, golden needle, shimeji, eryngii, and hen of the woods, produced in different areas of Japan in each mushroom category, were purchased at retail stores on three different days (Figure 1a–c). After placing these mushrooms into 2 L sample bags (Flek-sampler, Omi Odor-Air Service Corporation, Shiga, Japan), the bags were then filled with dry nitrogen (G1 grade), and allowed to equilibrate for 30 min (generally equilibration was needed for 15–60 min in the 2-L sample bag depending on the smell intensity) with the mushrooms still remaining in the sample bags (Figure 1d). Dry nitrogen was adopted to reduce the interference of moisture.

Champignon and truffle flavors from Le Nez Du Vin (Editions Jean Lenoir, Provence, France), a collection of flavors used for learning wine aromas, were used. To adjust the strength of its aroma to the measurement range of the sensors, 5 μL of the truffle flavor or 5 μL of the champignon flavor were left for 30 min in a 2-L sample bag filled with the dry nitrogen until the flavor reached equilibrium. For the champignon flavor, after leaving it for 30 min in a 2-L sample bag, the equilibrated gas was diluted to 1/25 concentration and then it was equilibrated by leaving it for another 30 min.

### 2.2. Sample Measurements

**Figure 2.**
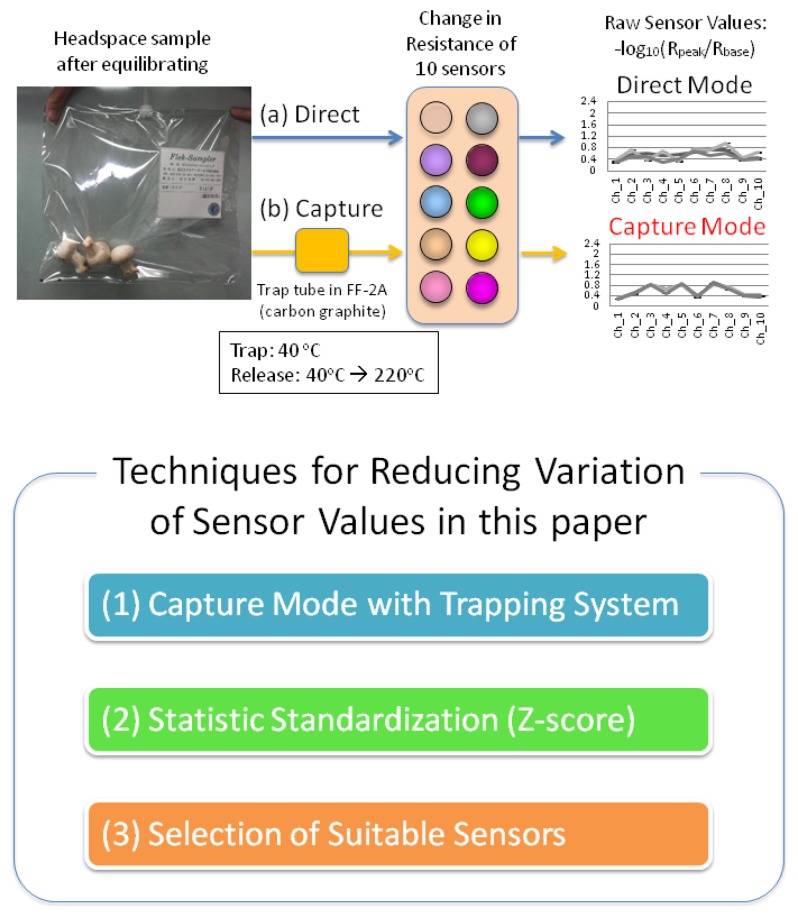
The measurement and analysis method scheme.

After the equilibrium, the FF-2A (Shimadzu Corporation, Kyoto, Japan), which is an odor recognition system equipped with 10 types of sensors with differing characteristics, was used to measure the samples. An outline of the measurement method using the FF-2A has been described in Figure 2 and previous reports [26–30]. Sampling was performed for 1 min at the rate of 165 mL/min from the sample bag. In the direct mode (Figure 2a), samples' volatiles were directly exposed to the sensors. On the other hand, in the capture mode (Figure 2b), after capturing samples components on a carbon graphite trap tube in the FF-2A for 30 s at 40 °C, heat-induced separation was performed by increasing to the temperature to 220 °C, and then the resistance values were measured. These sampling rate and temperatures were default setting for optimal measurement condition for sensors in FF-2A. Measurements were repeated four times for each sample. The six kinds of mushroom samples were measured on 1 day (in each trial) and repeated three times with 18 mushrooms on three different days (total 18 mushrooms = 6 mushrooms × 3 trials).

### 2.3. Analysis Methods

From the four measurement values, the values from the 2nd to 4th measurements were used for analysis. These measurement values from 10 types of sensors were standardized in each mushroom sample with formula (1) using Microsoft Office Excel 2007 (Microsoft Corporation, Redmond, WA, USA):

(1) $$\text{Z}=(\text{x}-\bar{\text{x}})/\text{s}$$

where Z: Standardized value (Z-score), x: Each sensor value, x̄: Average of 10 types of sensor values, s: Standard deviation for the 10 types of sensor values

Also, coefficients of variation (C.V.) were calculated with formula (2):

(2) $$\text{C}.\text{V}.=\text{s}/\bar{\text{x}}$$

For statistical analysis, PASW Statistics 17 (IBM, Armonk, NY, USA) was used to perform PCA (varimax rotation) and to calculate the Pearson correlation coefficients (*r*).

## 3. Results

### 3.1. Control of Variation with Capture Mode

**Figure 3.**
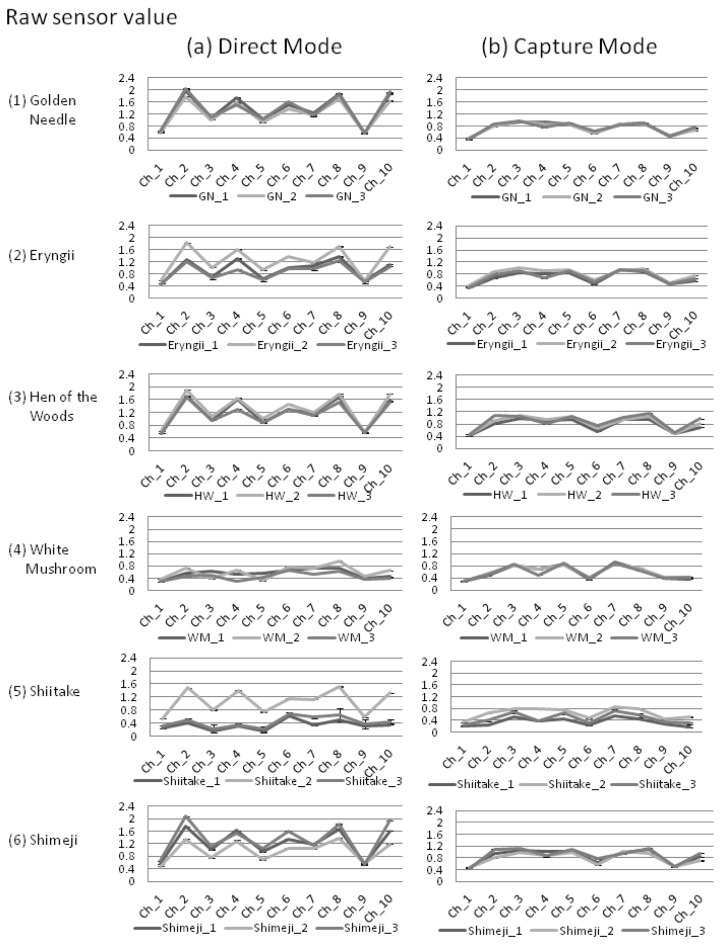
Electrical resistance value data for each sample using the two types of measurement methods, direct mode (**a**) and capture mode (**b**).

In this study, for the discrimination of shiitake, white mushrooms, golden needles, shimeji, eryngii, and hen of the woods mushrooms, the volatiles of these mushrooms were measured in two modes. In the first mode, volatiles were directly contacted with the 10 types of semiconductor sensors (direct mode: Figure 3a), and in the second mode, the volatiles were separated through heating after captured in a carbon graphite at 40 °C to reduce the influence of water and alcohol content and contacted the sensors (capture mode: Figure 3b).

For the sensor values, with the exception of white mushrooms and two varieties of shiitake (shiitake 1 and 3), we observed a tendency in the direct mode to exhibit higher values than capture mode (Figure 3). Also, in the direct mode, even among mushrooms of the same variety, differences greater than 0.8 were observed in the average of the sensor values (eryngii and shiitake).

The variation in each mode was reviewed using the coefficient of variation (C.V.) for the sensor values and the variations in *r*. As a result, the measured values were smaller for 75% of the C.V. among sensor values (45/60 sensor values) in the capture mode, and the average of C.V. for all sensor values were also smaller (Table 2).

The standard deviation values for the *r* between mushrooms of the same variety and between mushrooms of different varieties were lower in the capture mode than in the direct mode by greater than 0.1 (*i.e.*, 0.115 = 0.228 − 0.113, Table 3; 0.123 = 0.219 − 0.096, Table 4). From these standard deviations mentioned above, variation of raw data was smaller in capture mode than in direct mode.

### 3.2. Control of Variation by Statistic Standardization

Even in capture mode, which showed a low variation, a difference greater than 0.5 was observed in the sensor values of the sensor values among mushrooms of the same variety in eryngii and shimeji varieties (Figure 3b). Therefore, we attempted to standardize the measurement values to reduce the difference in the average values. For all samples, statistic standardization was used to produce z-score (average of values: 0, standard deviation: 1) for the 10 types of sensor values in each sample.

After standardization, the difference in the average z-score for both the mushrooms of the same variety and of different varieties was 0.055 (= 0.935 − 0.880) in the capture mode, which is greater than 0.027 (= 0.911 − 0.884) before standardization (Tables 3 and 4). On the other hand, in the capture mode, the standard deviation for the *r* increased by 0.006 (= 0.102 − 0.0096) for mushrooms of different varieties (Table 4) and decreased by 0.091 (= 0.113 − 0.042) for mushrooms of the same variety (Table 3). In a similar manner, in the direct mode, the difference between average values for the *r* increased, and the standard deviation among mushrooms of the same variety decreased. From these results, it was shown that through the statistic standardization: (1) the differences among average values of all samples were eliminated, because the average sensor values from all samples converged to 0; (2) the *r* for mushrooms of the same variety increased; and (3) variation was decreased in the *r* for mushrooms of the same variety.

### 3.3. Discrimination Based on the Sensor Values Suitable for Screening

**Figure 4.**
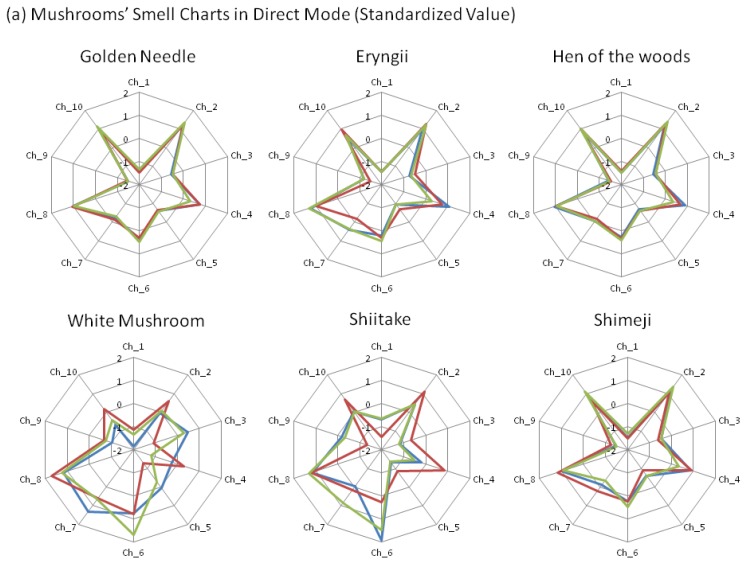

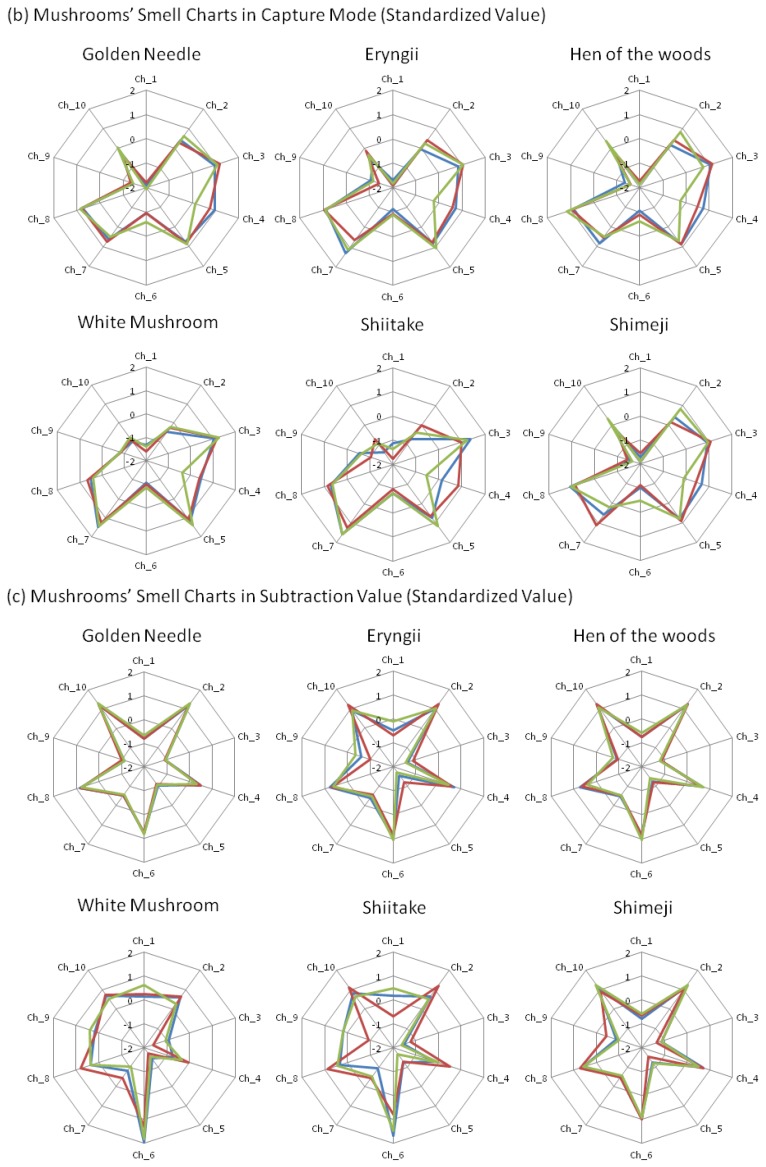
Standardized values in (**a**) direct mode; (**b**) capture mode; (**c**) subtraction values. (Blue: 1st trial, Red: 2nd trial, Green: 3rd trial.)

With the attention focused on individual charts in capture mode, the chart patterns between the white mushrooms and shiitake, as well as those between four other mushroom varieties, were considered to be similar to each other (Figure 4b). In addition, the average value for the *r* between these four mushroom varieties exceeded 90% (data not shown). In other words, in the capture mode, the chart shapes for the two mushroom groups belonging to white mushrooms and shiitake have shown a tendency to differ from those of the other four mushroom groups.

Next, upon observation of the individual z-scores, certain characteristics were noted in each mushroom. For shiitake and white mushrooms, the z-score of Ch_7 was greater than 1.2 and the score of Ch_10 was smaller than 0.7 (Figure 4b). With white mushrooms in particular, the score of Ch_8 tended to be under 0.7, suggesting that it was possible to discern shiitake and white mushrooms. Furthermore, eryngii exhibited characteristics in which (1) the score of Ch _7 exceeds 1 and the score of Ch_9 is greater than −1; or (2) the score of Ch_7 is less than 0.7 and Ch_9 is greater than −1.5.

On the other hand, in the direct mode, variations in the z-scores for white mushrooms and shiitake were observed even among mushrooms of the same variety (Figure 4a). For the values obtained by subtraction focusing on the difference in values between the direct mode and capture mode (*i.e.*, differences caused by polar components such as water, alcohol, *etc.*), golden needle exhibited the characteristics that the z-score of Ch_9 was less than 1 and Ch_10 exceeded 1.2 in subtraction value (Figure 4c).

**Figure 5.**
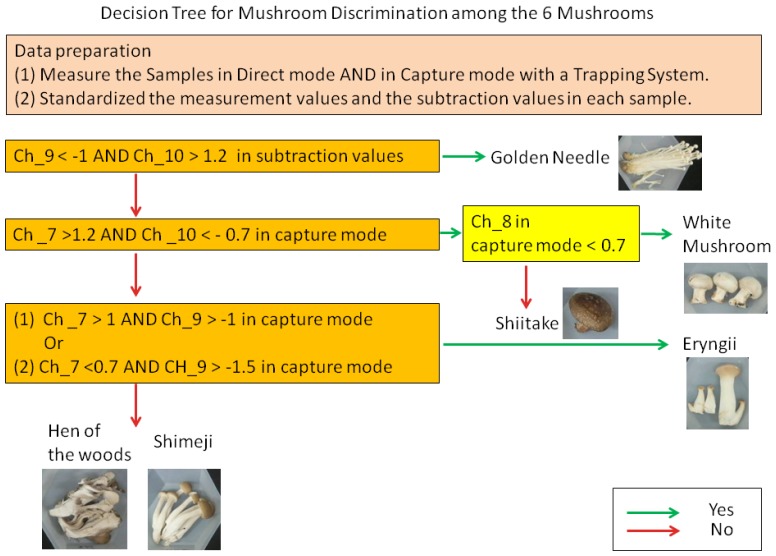
A decision tree for categorizing four out of the six mushroom varieties using z-scores.

These characteristic scores suitable for screening in Ch_7–10 were applied to two methods for discrimination, a decision tree and PCA. First, we structured a decision tree for mushrooms discrimination that used z-scores in capture mode and subtraction (Figure 5). Using this tree, four varieties (golden needles, eryngii, shiitake, and white mushrooms) among six mushrooms varieties were discriminated.

**Figure 6.**
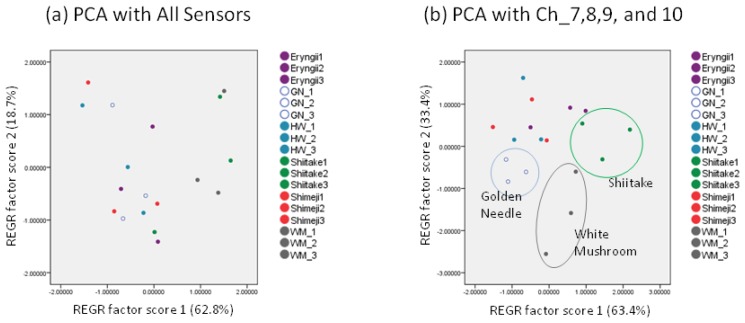
Mushroom discrimination by principal component analysis. (**a**) Principal component analysis using all sensor values; (**b**) principal component analysis using Ch_7–10.

Next, the PCA results obtained from the capture mode values are shown in Figure 6. Although it was not possible to discriminate mushrooms with PCA using all sensor scores (Figure 6a), using the scores of Ch_7–10, it was possible to discriminate golden needles, white mushrooms, and shiitake (Figure 6b).

### 3.4. Discrimination with Artificial Mushroom Flavors

**Figure 7.**
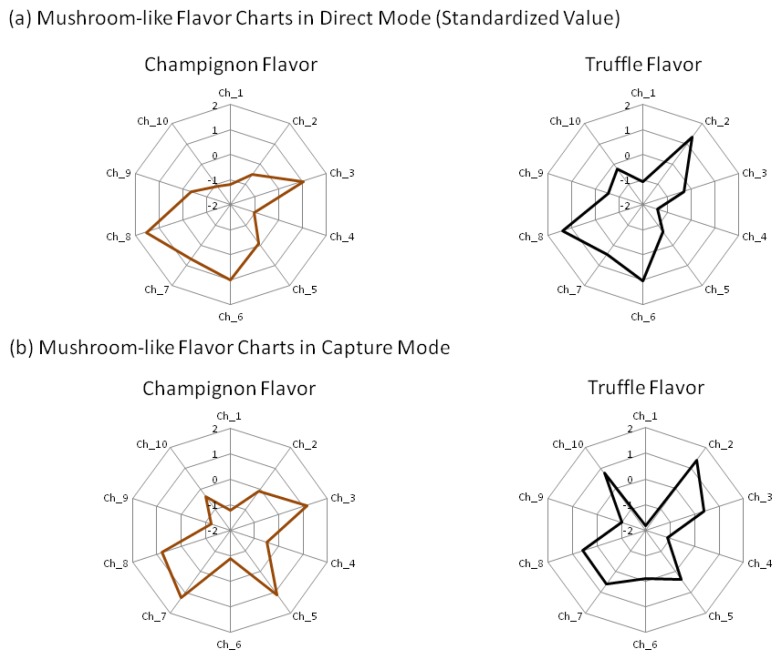
Standardized values for two types of mushroom flavors, champignon flavor and truffle flavor in (**a**) Direct mode and (**b**) capture mode.

Finally, we investigated the discrimination technique using correlation for two types of artificial mushroom flavors (champignon flavor and truffle flavor). After standardization of the flavors' sensor values (Figure 7), the *r* for the flavors and mushroom samples was calculated (Table 5).

In direct mode, all mushrooms had a high correlation with the champignon flavor compared with the truffle flavoring, while in capture mode, many mushrooms tended to have a higher *r* with the champignon flavor. In capture mode for the white mushrooms, the *r* with the champignon flavor were greater than 0.916 and with the truffle flavor less than 0.493, which showed characteristics not found in other mushrooms. Therefore *r* of the white mushrooms with the champignon flavor and truffle flavor exhibited the patterns that differ from other mushrooms.

## 4. Discussion

This study demonstrated that in the discrimination of fresh mushrooms using sensors, the capture mode, wherein the influence of water and alcohol content has been reduced, and the statistical standardization were effective for control of data variation. Moreover, it was shown that through the use of sensor values suitable for screening, it is possible to discriminate four mushroom varieties with decision tree (golden needles, eryngii, shiitake, and white mushrooms) and three mushroom varieties with PCA (golden needles, shiitake, and white mushrooms) among six mushroom varieties.

Generally, variations in the values measured by electronic noses are attributed to interferences (e.g., humidity), drift effects due to sensor deterioration [31], ambient temperature, the use of different filters, and changes in mass flow rates [32]. Particularly, humidity has a large impact on measurement results; hence, the construction of a calibration model for improvement [33], the construction of a system to control baseline humidity within a suitable range [34], or the introduction of pre-filters [22] have been conducted.

Since fresh mushrooms contain large amounts of water and alcohol, several gas sensors are affected by these components. Moreover, these components themselves are unstable, causing variations that affect the accuracy of discrimination. In fact, in the previous study, classification of production area using an electronic nose was difficult due to variations in the measurement values [21], and this phenomenon has also been observed in our direct mode data (Figure 3a).

Therefore, in this study, we introduced two methods to reduce variations. Firstly, in the capture mode method, the carbon graphite trap tube was used to control the influence of water and alcohol contents. Through this method, we showed that it was possible to control the variation for mushrooms of the same variety in comparison to the direct measurement method (Figure 3b). This trend was prominent in the shiitake, shimeji, and eryngii. Secondly, the statistic standardization method enables the measurements to be compared with each other easily. Using this technique, differences among the average of sensor values for mushrooms were eliminated, while the *r* between mushrooms of the same variety was improved by 0.024 and the standard deviation decreased by 0.091 (Table 3). In addition to these variation control methods, by selecting the sensor values, we were able to discriminate four varieties out of six mushroom varieties with the decision tree (Figure 5), and with PCA, it was possible to discriminate to three mushroom varieties (Figure 6).

On the other hand, these methods employed to control the variation have technical limitations. In the capture mode, information on polar components (such as water, alcohol, and components that dissolve in these contents) was almost removed, so it may be difficult to perform discrimination with mushroom varieties rich in these components. In these rich polar components samples, it may be effective to utilize discrimination in which the difference is taken between the direct mode and capture mode used in this study (Figure 4c), or it may be effective to use the humidity controlling system in other studies [34]. Additionally, in the statistical standardization method, the intensity information of the total aroma is lost, while the quality information of aroma remains. As a result, this method is suitable for discrimination purposes but not for examination of aroma intensity. For these reasons, when applying the method used in this study to control variation, it is necessary to consider the aroma characteristics and the intended information.

We discuss the usefulness of this study in the three sensor applications listed in the Introduction section. First, this study may lead to preventing food poisoning. Our results showed that it is possible to discriminate four varieties among six mushroom varieties to reduce the variation. In the future, it may be useful to investigate a comparison of the aromas between edible mushrooms and easily-confused toxic mushrooms. To determine the edibility of mushrooms, an algorithm were reported that uses 19 rules based on the information of mushroom features in a Mushroom Data Set, UCI [35], such as aroma, color, *etc.* [36]. This algorithm may not reflect real mushrooms directly, since this data set contains virtual data. However, if the objective aroma data were collected with electronic noses, it may lead to determine whether mushrooms discovered in fields were edible.

Next, we discuss possibilities in relation to consumer information by constructing a data set that contains data from sensor-based objective evaluation of aromas. In this study, to obtain mushroom aroma information, the similarity to two types of mushroom flavors was calculated (Table 5). From the *r* value in the capture mode, white mushrooms showed higher correlation with the champignon flavor and lower correlation with truffle flavor. These results suggested that white mushrooms have an aroma that was close to champignon flavor and distant to truffle flavor, compared with other mushrooms. Therefore, we can score the distances of the aroma between mushrooms and flavors using electronic noses.

In contrast to our sensor techniques, another study showed the scores about the features of mushrooms with the human senses of smell and taste [37]. This study reported that mushroom-like aromas trigger a negative impression in some consumers; hence, mushrooms that have lower score than other mushrooms have an advantage in the food industry. Additionally, there may be different aroma preferences in Europe and Japan, such as those of shiitake and other such mushrooms aromas [38].

In our study, no discrimination was performed by human senses, so in the future, comparisons with the human senses will be necessary. With regards to the comparison and adjustment of sensor values to human senses, Williams *et al.* have reported a method in which electronic nose values and a neutral network were applied to predict pleasant/unpleasant score judged by humans [39]. Hence, it may be possible to predict the mushroom-like aroma score or the pleasant/unpleasant score in human senses, by implementing the similar adjustment method of the electronic nose.

Finally, to develop the mushroom discovery devices, it may be effective to provide electronic noses with memory of characteristic aromas such as real fresh mushroom or mushroom flavors. Truffles contain 5α-androst-16-en-3α-ol [40], which is considered to be one of the primary smell components among the volatiles of many truffles. Additionally, truffles contain dimethyl sulfide [41], which may be used as hints for truffle searches by dogs and pigs [42]. A previous study has reported that a hydrogen flame ionization detector (FID)—a type of gas sensor—is useful in discovering truffles [43]. However, a compact electronic nose that has learned characteristic truffle aromas such as 5α-androst-16-en-3α-ol and dimethyl disulfide, or sensors suitable for these aromas, may discover truffles easier.

## 5. Conclusions

In this study, we discriminated four fresh mushrooms among six fresh mushrooms by performing the following steps: (1) using a trapping system to reduce inference components; (2) statistic standardization (calculation of z-score); and (3) selecting suitable sensors. Additionally, comparison to artificial mushroom flavors was useful in discriminating white mushroom. Our techniques will open up the option to reduce variation in sensor values.

## Figures and Tables

**Table 1.** Main Volatile Components of Mushrooms in References.

**General Name**	**Scientific Name**	**Sample Condition: Main Components**	**Reference**
Golden Needle	*Flammulina velutipes*	Fresh: Ethanol, isobutanol, methyl acetate, acetone, ethyl acetate, isobutanal	Imai *et al.* (1999) [12]
Eryngii	*Pleurotus eryngii*	Fresh: 3-Octanone, 1-octen-3-one, 3-octanol, 1-octen-3-ol, benzaldehyde, 1-octanol, 2-octen-1-ol	Mau *et al.* (1998) [13]
Hen of the Woods	*Grifola frondosa*	Frozen: 1-Octen-3-ol, 3-methylbutan-2-one, methyl 2,4-dihydroxybenzoate, heptene, δ-cadinene, benzyl alcohol, benzaldehyde	Rapior *et al.* (1996) [14]
White Mushroom	*Agaricus bisporus*	Frozen: 1-Octen-3-ol, benzyl alcohol, hexanal, benzaldehyde, (*E,Z*)-2,4-decadienal, (*E,E*)-2,4-decadienal	Rapior *et al.* (1996) [14]
		Fresh: 1-Octen-3-ol, 3-octanol, 2*Z*-octenol, 3-octanone	Venkateshwarlu *et al.* (1999) [15]
Shiitake	*Lentinus edodes*	Fresh: 1-Octen-3-ol, 3-octanone, dimethyl disulfide, dimethyl trisulfide	Wu *et al.* (2000) [16]

**Table 2.** Coefficient of Variation (C.V.) in direct mode and capture mode (n = 3).

	**Ch_1**	**Ch_2**	**Ch_3**	**Ch_4**	**Ch_5**	**Ch_6**	**Ch_7**	**Ch_8**	**Ch_9**	**Ch_10**	**Average**
GN_Direct	0.127	0.221	0.203	0.146	0.234	0.198	0.062	0.158	0.013	0.253	0.162
GN_Capture	0.077	0.103	0.074	0.065	0.060	0.105	0.057	0.053	**0.029**	0.119	0.074
Eryngii_Direct	0.463	0.820	0.700	0.858	0.723	0.458	0.562	0.634	0.292	0.867	0.638
Eryngii_Capture	0.419	0.629	0.335	0.472	0.387	**0.486**	0.285	0.442	0.285	0.745	0.448
HW_Direct	0.042	0.033	0.037	0.020	0.044	0.036	0.019	0.018	0.014	0.040	0.030
HW_Capture	**0.102**	**0.089**	**0.071**	**0.049**	**0.084**	**0.084**	**0.050**	**0.091**	**0.018**	**0.110**	**0.075**
WM_Direct	0.177	0.334	0.360	0.346	0.368	0.201	0.215	0.222	0.106	0.342	0.267
WM_Capture	**0.261**	0.201	0.115	0.120	0.129	0.158	0.097	0.139	0.067	0.253	0.154
Shiitake_Direct	0.157	0.262	0.233	0.237	0.265	0.241	0.108	0.210	0.020	0.296	0.203
Shiitake_Capture	**0.170**	0.178	0.073	0.109	0.077	0.164	0.087	0.143	**0.055**	0.210	0.126
Shimeji_Direct	0.466	0.898	0.736	0.951	0.777	0.544	0.457	0.665	0.228	0.942	0.666
Shimeji_Capture	0.339	0.543	0.253	0.440	0.235	0.476	0.118	0.381	0.156	0.633	0.358

*Note*: **Bold**: The value in capture mode was higher than direct mode. GN: Golden needle; HW: Hen of the wood; WM: White mushroom.

**Table 3.** Average of Correlation Coefficient (*r*) against “Same Mushrooms” (*ex*. Eryngii_1 *vs.* Eryngii_2…).

	**Raw Data**		**Standardization Data (Z-score)**	

	**Direct (n=18)**	**Capture (n=18)**	**Direct (n=18)**	**Capture (n=18)**
Average	0.811	0.911	0.882	0.935
Standard Deviation	0.228	0.113	0.153	0.042

**Table 4.** Average of Correlation Coefficient (*r*) against “Other Mushrooms” (*ex*. Eryngii_1 *vs.* Shiitake_1…).

	**Raw Data**		**Standardization Data (Z-score)**	

	**Direct (n=135)**	**Capture (n=135)**	**Direct (n=135)**	**Capture (n=135)**
Average	0.766	0.884	0.771	0.880
Standard Deviation	0.219	0.096	0.223	0.102

**Table 5.** Correlation Coefficient (*r*) between Mushrooms' Smells and Mushroom-like Flavors using Standardized Sensors' Values of Ch_7–10.

	**Champignon Flavor**				**Truffle Flavor**				

	**Direct Mode**	**Capture Mode**	**Both Mode**	**Subtraction**	**Direct Mode**	**Capture Mode**	**Both Mode**	**Subtraction**
GN_1	0.178	0.95	0.523	−0.356	0.593	0.874	0.717 *	−0.611
GN_2	0.229	0.982 *	0.576	−0.325	0.634	0.793	0.706	−0.601
GN_3	0.123	0.899	0.468	−0.347	0.547	0.914	0.709 *	−0.632

Eryngii_1	0.525	0.987 *	0.763 *	−0.233	0.84	0.637	0.733 *	−0.509
Eryngii_2	0.202	0.942	0.549	−0.322	0.612	0.866	0.732 *	−0.624
Eryngii_3	0.461	0.996 **	0.727 *	−0.065	0.802	0.753	0.776 *	−0.454

HW_1	0.27	0.981 *	0.611	−0.312	0.666	0.792	0.727 *	−0.607
HW_2	0.173	0.919	0.528	−0.332	0.589	0.882	0.729 *	−0.655
HW_3	0.175	0.828	0.501	−0.288	0.59	0.941	0.767 *	−0.679

WM_1	0.857	**0.916**	0.886 **	0.484	0.876	**0.431**	0.659	−0.083
WM_2	0.79	**0.947**	0.858 **	0.264	0.979 *	**0.493**	0.711 *	−0.119
WM_3	0.908	**0.931**	0.916 **	0.679	0.982 *	**0.489**	0.715 *	0.15

Shiitake_1	0.723	0.829	0.746 *	0.45	0.882	0.246	0.428	−0.134
Shiitake_2	0.389	0.980 *	0.684	−0.322	0.754	0.604	0.678	−0.570
Shiitake_3	0.894	0.897	0.866 **	0.437	0.985 *	0.382	0.597	−0.100

Shimeji_1	0.229	0.913	0.566	−0.322	0.634	0.877	0.755 *	−0.654
Shimeji_2	0.429	0.996 **	0.723 *	−0.122	0.781	0.785	0.780 *	−0.458
Shimeji_3	0.092	0.771	0.415	−0.323	0.52	0.934	0.718 *	−0.664

*Note*: Correlation is significant at the 0.01

** or 0.05

* level (2-tailed);

**Bold**: Distinguishing values in white mushrooms
